# En bloc underwater endoscopic mucosal resection of a large laterally spreading tumor in the colon after endoscopic tattooing

**DOI:** 10.1055/a-2445-8524

**Published:** 2024-11-08

**Authors:** Xing Hua Ma, Kengo Kasuga, Ayaki Isshiki, Shingo Ishihara, Takashige Masuo, Yoji Takeuchi, Toshio Uraoka

**Affiliations:** 1 37002 Department of Gastroenterology, Isesaki Municipal Hospital, Isesaki, Japan; 2 Department of Gastroenterology and Hepatology, Gunma University Graduate School of Medicine, Maebashi, Japan

Underwater endoscopic mucosal resection (UEMR) is an alternative technique to standard polypectomy for managing complex cases. The primary advantage of UEMR is that it causes less bowel lumen distension, potentially allowing the capture of a larger mucosal surface area 1 .

A 64-year-old woman was referred to our hospital after colonoscopy revealed cecal cancer and a 25-mm laterally spreading tumor (LST) in the transverse colon, diagnosed as intramucosal cancer ( Fig. 1 **a, b** ). Initially, we thought that both the transverse colon and the cecal tumor would need to be removed surgically, leading to the tattooing of the anal side of the LST ( Fig. 1 **c** ). However, endoscopic resection of the LST in the transverse colon would reduce the invasiveness of surgery. Therefore, we chose UEMR as the method for en bloc resection of the LST ( Fig. 1 **d, e** , Video 1 ).

**Fig. 1:**
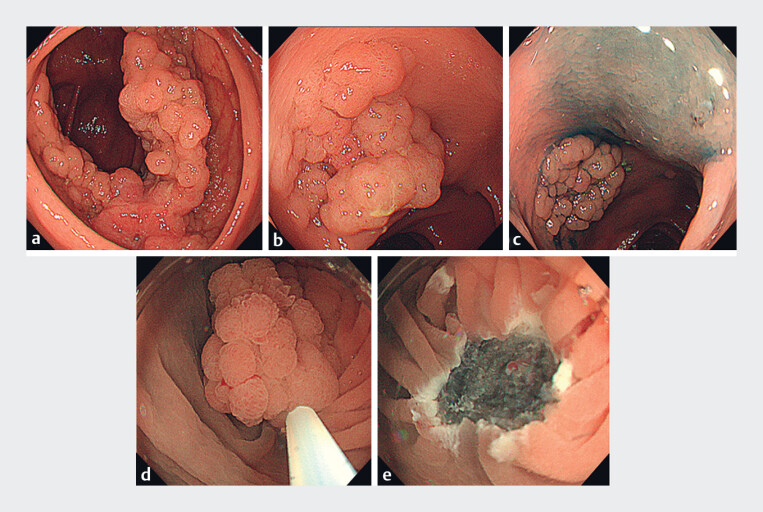
Endoscopy images. **a** An invasive cancer in the cecum. **b** A 25-mm laterally spreading tumor (LST) located in the transverse colon. **c** Tattooing at the anal side of the LST. **d** Underwater endoscopic mucosal resection (UEMR) of the LST. **e** Mucosal defect after UEMR, showing black-colored submucosal tissue.

Tattooing and en bloc removal of a laterally spreading tumor in the transverse colon with underwater endoscopic mucosal resection.Video 1

The LST was resected en bloc using an extra-large snare (33 mm, Captivator II; Boston Scientific, Marlborough, Massachusetts, USA) ( Fig. 2 **a, b** ). Histopathological examination revealed an intramucosal adenocarcinoma with adenoma, without lymphovascular involvement and no tumor involvement of the resection margins ( Fig. 2 **c, d** ).

**Fig. 2:**
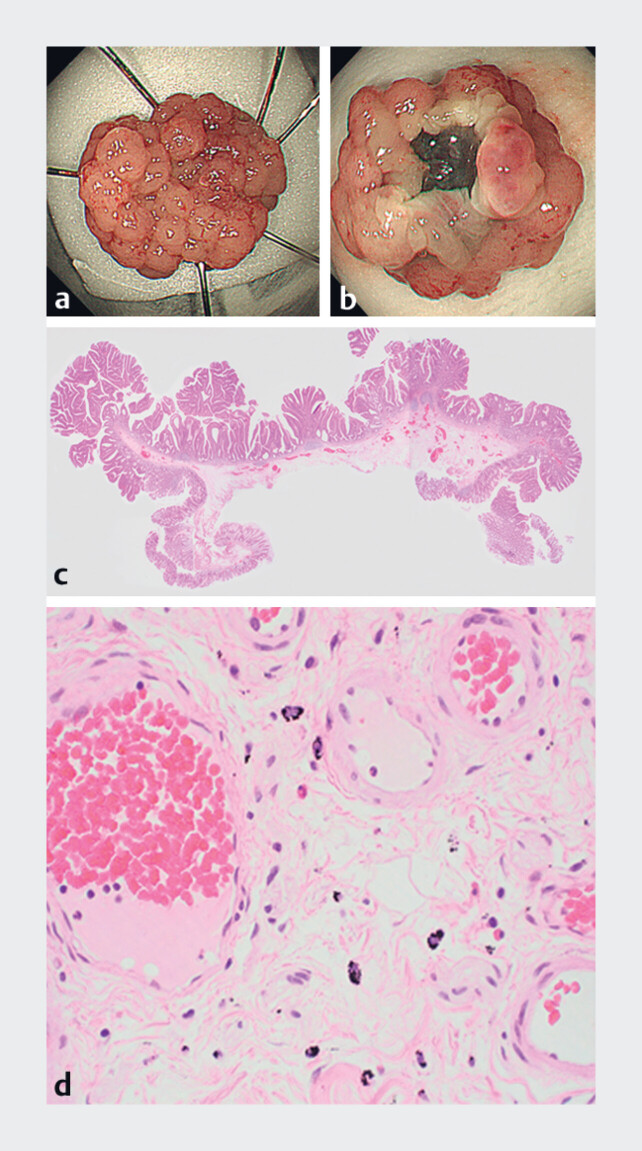
Findings from the resected tumor. **a** En bloc resected specimen. **b** The submucosal side of the lesion, showing non-neoplastic mucosa around the lesion. **c** Microscopic image of the resected specimen indicating an intramucosal adenocarcinoma with adenoma, without lymphovascular involvement and no tumor involvement of the resection margins. **d** Macrophages containing carbon pigments in the submucosal layer.

After endoscopic tattooing for colorectal lesions, endoscopic submucosal dissection (ESD) is challenging due to fibrosis and unclear anatomical layers in the submucosal layer 2 . Conversely, UEMR has shown its effectiveness in treating local residual/recurrent colorectal lesions, suggesting its usefulness for lesions with fibrosis 3 . In a previous report, UEMR was performed in a piecemeal manner for a tattooed tumor 4 . However, UEMR for 20–30-mm lesions is reportedly comparable to ESD in terms of local recurrence 5 . In the current case, even a 25-mm lesion could be resected en bloc with UEMR, resulting in precise pathological assessment. This case demonstrates the advantage of UEMR for a large LST even after endoscopic tattooing.

Endoscopy_UCTN_Code_TTT_1AQ_2AD_3AC

## Notes

Endoscopy E-Videos https://eref.thieme.de/e-videos
*E-Videos* is an open access online section of the journal *Endoscopy* , reporting on interesting cases and new techniques in gastroenterological endoscopy. All papers include a high-quality video and are published with a Creative Commons CC-BY license. Endoscopy E-Videos qualify for HINARI discounts and waivers and eligibility is automatically checked during the submission process. We grant 100% waivers to articles whose corresponding authors are based in Group A countries and 50% waivers to those who are based in Group B countries as classified by Research4Life (see: https://www.research4life.org/access/eligibility/ ). This section has its own submission website at https://mc.manuscriptcentral.com/e-videos .
